# Maternal attachment and perception of motherhood in relation to third-trimester antenatal depression: a cross-sectional analysis

**DOI:** 10.25122/jml-2025-0143

**Published:** 2025-10

**Authors:** Nicoleta Soldan, Cristiana Glavce, Andrei Kozma, Cristina Stan, Monica Petrescu, Roxana Maier, Suzana Turcu

**Affiliations:** 1 Medical Anthropology, Francisc I Rainer Institute of Anthropology, Bucharest, Romania; 2 Research Department, Alessandrescu-Rusescu National Institute for Mother and Child Health, Bucharest, Romania; 3 Faculty of Psychology, Ecological University of Bucharest, Bucharest, Romania

**Keywords:** antenatal depression, Edinburgh Postnatal Depression Scale, adult attachment, perception of motherhood, screening, third trimester, AAS, Adult Attachment Scale, ACOG, The American College of Obstetricians and Gynecologists, aOR, adjusted odds ratio, CI, confidence interval, EPDS, Edinburgh Postnatal Depression Scale, NICE, The National Institute for Health and Care Excellence, R-AAS, Revised Adult Attachment Scale, USPSTF, The US Preventive Services Task Force, WHO, World Health Organization

## Abstract

Antenatal depression is a common complication of pregnancy, with consequences spanning maternal mental health, obstetric outcomes, and early mother–infant adaptation. Effective early identification requires integrating psychological and contextual information alongside validated screening. This study examined whether adult attachment style and the perception of motherhood are associated with antenatal depressive severity in late pregnancy, beyond socio-demographic factors. In a cross-sectional analysis of 140 third-trimester women, adult attachment (Revised Adult Attachment Scale, R-AAS) and depressive symptoms (Edinburgh Postnatal Depression Scale, EPDS) were assessed together with psychosocial indicators (pregnancy planning, partner support, and perception of motherhood). Bivariate associations were tested with χ^2^ (Cramér’s V), and multivariable effects with penalized logistic regression for EPDS ≥14, using bootstrap 95% CIs (B = 1000). Secure attachment was associated with minimal risk (0% EPDS ≥14), whereas anxious–ambivalent attachment showed increased vulnerability (49.4% EPDS ≥12). A negative perception of motherhood displayed the most severe profile (60.0% EPDS ≥14 vs 0% in the positive group). In adjusted models, negative perception (aOR = 21.07; 95% CI, 7.92–1317.40) and anxious–ambivalent attachment (aOR = 21.67; 95% CI, 1.00–77.96) retained independent associations, while other covariates were not significant. These findings support a pragmatic psychosocial screening approach for late pregnancy in which a single standardized question on the perception of motherhood and a brief attachment typology add clinically useful information to EPDS. Incorporating these elements into routine antenatal care may enhance early detection and facilitate timely referral to perinatal mental-health services, with multicentre validation needed to support wider implementation.

## Introduction

Antenatal depression is one of the most common complications of pregnancy and is associated with affective symptoms, suicidal risk, and adverse obstetric outcomes (preterm birth, low birth weight, breastfeeding difficulties), with downstream effects on child development and the mother–infant dyad [1-6]. Beyond immediate clinical sequelae, persisting symptoms may interfere with bonding, nurturing behaviors, and the quality of early caregiving, with potential reverberations across infancy and the preschool years [5,6]. Recent systematic reviews and meta-analyses estimate the prevalence of depressive symptoms in pregnancy at 20–30%, although figures vary by gestational timing, assessment instruments, and socio-economic context, including low-and middle-income settings [1-4]. Taken together, these observations position perinatal mental health as a core component of obstetric, primary care, and public health strategies [5,6].

In clinical practice and population health, major bodies recommend routine screening for depression and anxiety across antenatal and postnatal care, embedded within clear referral and treatment pathways. The American College of Obstetricians and Gynecologists (ACOG) recommends screening at the first prenatal visit and at least once later in pregnancy and postpartum [7]. The National Institute for Health and Care Excellence (NICE) CG192 guideline sets out the identification and management of perinatal mental disorders [8], while the World Health Organization (WHO) advocates integrating perinatal mental health within maternal and child health services [9]. Complementing detection, the US Preventive Services Task Force (USPSTF) recommends offering preventive psychological interventions (e.g., cognitive behavioral therapy, interpersonal therapy) to those at increased risk [10]. Across these frameworks, screening has value only where referral pathways are functional and treatment capacity exists.

Perinatal affective disorders represent a significant public health issue, demonstrating considerable prevalence and well-documented effects on mothers, infants, and the mother-infant relationship [1-3]. Accordingly, the use of validated screening instruments, such as the Edinburgh Postnatal Depression Scale (EPDS), is essential for identifying perinatal affective vulnerability and for guiding appropriate care pathways. Beyond symptom counts, proximal psychosocial factors shape perinatal adaptation, with adult attachment styles and representations of motherhood playing central roles. Recent syntheses indicate that prenatal maternal attachment and perinatal depressive symptomatology are generally inversely associated, while positive representations of motherhood correlate with lower depressive symptoms and more favorable affective adjustment [6-8]. These links are context-dependent: relational (e.g., partner support) and cultural environments influence both direction and magnitude, including in Eastern European settings [3,11]. In Romania, socio-cultural scholarship highlights how family norms and cultural scripts of motherhood shape emotional expression, readiness to seek support, and role expectations—factors relevant to attachment and perinatal affective risk [12].

Attachment theory offers an explanatory framework for inter-individual variability in affect regulation, perceived support, and stress processing during pregnancy. In adults, secure, avoidant, and anxious–ambivalent patterns are associated with distinct coping strategies and differential affective vulnerability; perinatal studies report links between relational insecurity and antenatal depressive symptoms, as well as downstream effects on postnatal bonding [11,13,14]. From a clinical standpoint, a brief adult attachment typology can flag heightened vulnerability to antenatal depressive symptoms and difficulties in early bonding, adding information beyond symptom scores. Plausible mechanisms include hyperactivation or deactivation of the attachment system, differences in mentalization capacity and expectations regarding others’ availability and responsiveness, which together inform appraisals of stressors and preferences for help-seeking in pregnancy.

Beyond psychometric constructs, modifiable psychosocial factors shape risk in clinically meaningful ways. Pregnancy intentionality and partner or wider social support show consistent associations with perinatal symptomatology [15-17]. Reproductive age, parity, educational level, and marital status recur as contextual correlates; incorporating these variables into assessment can improve risk estimation and prioritize timely referral [1–6,12,18,19]. This integrative approach aligns with trends towards personalized care and early, profile-tailored interventions within obstetric services.

In Central-Eastern Europe, heterogeneity in reported prevalence, instruments, and associated factors reflects contextual differences and methodological diversity. Nevertheless, local data support the use of EPDS and delineate a plausible risk profile for Romania, strengthening the case for context-adapted screening protocols [6,18]. The third trimester is particularly salient: neuroendocrine reorganization and psychosocial demands peak before birth, and clinical decisions (including referral to specialized services) can have immediate perinatal consequences. Practically, late pregnancy is an actionable window to embed rapid psychosocial assessment into birth planning and the immediate postpartum period.

We therefore sought, in the third trimester, to articulate a pragmatic psychosocial risk profile for antenatal depression by integrating information from three complementary sources: adult attachment style measured with the Revised Adult Attachment Scale (R-AAS), the perception of motherhood (positive, ambivalent, or negative) and socio-demographic characteristics (age, parity, educational level, marital status), with the addition of pregnancy planning and partner support. We hypothesized that anxious–ambivalent attachment and a negative perception of motherhood would be associated with higher EPDS severity, whereas secure attachment and favorable contextual factors would be protective, thereby informing screening and referral in routine antenatal care [7-10].

## Material and Methods

### Study design and setting

We conducted a cross-sectional, observational analysis of third-trimester pregnant women in urban Romania. Recruitment drew on prenatal clinical networks, private medical practices, and community outreach in Bucharest and other cities. The design permits contemporaneous estimation of associations between psychosocial factors and antenatal depressive severity in late pregnancy.

### Participants and recruitment

A total of 140 third-trimester women were enrolled based on availability and interest via the recruitment routes above, which broadened socio-cultural coverage and enhanced feasibility. Inclusion criteria were adult age, singleton viable pregnancy, adequate literacy, and provision of written informed consent. Exclusion criteria targeted conditions that would impede questionnaire completion or introduce acute clinical risk at assessment. No additional exclusions were applied.

### Procedure and data collection

Questionnaires were administered individually, with investigator support, under conditions of confidentiality. Depending on preference and practical constraints, the work was completed on paper or in a secure digital format. Average administration time was approximately 30–35 minutes. Participants received information about the aims and limits of the study, confidentiality safeguards, and the exclusively scientific use of data. Safety procedures were in place for item-level triage: EPDS item 10 (suicidal ideation) was monitored, with information and referral according to protocol when indicated.

### Measures

Attachment was assessed using the Revised Adult Attachment Scale (R-AAS; derived from the Adult Attachment Scale, Collins & Read, 1990), yielding continuous scores on closeness (comfort with intimacy), dependence (trust/reliance on others), and anxiety. The R-AAS comprises 18 items rated on a 5-point Likert scale (1 = not at all characteristic, 5 = very characteristic), grouped into three six-item subscales: closeness, dependence, and anxiety [20]. Higher closeness and dependence scores indicate comfort with intimacy and trust, whereas higher anxiety reflects fear of rejection or abandonment.

For each participant, mean scores on the three dimensions were standardized within the sample (z-scores). Cut-offs were set at the sample median. A priori decision rules were applied as follows: (i) **secure** – anxiety < median and at least one of closeness or dependence ≥ median; (ii) **avoidant** – closeness < median and dependence < median in the absence of elevated anxiety; (iii) **anxious–ambivalent** – anxiety ≥ median (classification priority). Where rules conflicted, the priority order was anxious–ambivalent > secure > avoidant. An exploratory tertile-based classification yielded similar distributions, supporting the robustness of this operationalization.

Internal consistency in the present sample was satisfactory (Cronbach’s α = 0.79 for closeness, 0.83 for dependence, and 0.86 for anxiety). The Romanian versions of both the R-AAS and the EPDS have demonstrated factorial validity and criterion correspondence with depressive symptomatology [20,21]. Perception of motherhood was assessed with a single standardized item: “How do you currently perceive motherhood for yourself?”, with pre-specified response options (positive, ambivalent, negative). Coding followed these categories. This single-item indicator was chosen for feasibility and clinical utility in obstetric settings, complementing the EPDS by capturing representations not reflected in symptom scores.

The EPDS remains the most widely used screening instrument; an individual participant data meta-analysis supports its diagnostic accuracy across multiple cut-offs and populations [22]. Short forms (e.g., EPDS-9) can be useful in resource-limited settings, as these offer comparable performance in some contexts, aiding rapid triage and repeated assessments in busy clinics [23]. In practice, EPDS offers a brief, acceptable, and scalable screener with well-characterized accuracy. However, scores should be interpreted alongside each woman’s psychosocial profile, as contextual factors can modulate risk, influence symptom expression, and shape help-seeking and response to intervention.

Although initially designed for postnatal use, the EPDS has been extensively validated for antenatal screening, including in the third trimester, where it maintains sensitivity and specificity for depressive symptomatology. We used it in this context as recommended by international perinatal mental health guidelines [24,25].

The EPDS scale (10 items; total 0–30) used study cut-offs: 0–8 (absent), 9–11 (possible), 12–13 (probable), ≥14 (screen-positive). Item 10 (suicidal ideation) was flagged for immediate review, with information and referral per protocol. EPDS was selected for its brevity, diagnostic performance, and ease of integration into obstetric workflows [11,12].

Socio-demographic characteristics included age, parity, educational level, and marital status. Pregnancy planning and partner support were additionally recorded to reflect modifiable risk contexts relevant to screening and referral. Coding followed the study protocol.

Assisted administration minimized missing data; questionnaires were checked on site, and omissions were completed immediately. No imputation was used. EPDS analyses required complete questionnaires (the ≥9/10 rule was not needed in practice). No exclusions were required due to missing data.

### Sample size and data quality

The analysis included *n* = 140 third-trimester participants. To mitigate sparse cells and potential (near) complete separation in multivariable models, we used L1-penalised logistic regression. Non-parametric bootstrap 95% confidence intervals (CIs) were computed with B = 1000 resamples (percentile method), using a fixed random seed for reproducibility. Completeness checks were performed after each administration; item non-response was minimal and did not require imputation. The sample size (*n* = 140) was determined pragmatically from the available recruitment period but exceeded the conventional 10 events per variable rule for logistic models and was comparable to prior perinatal psychosocial studies in Eastern Europe [6,19].

### Endpoints and analytical strategy

The primary endpoint was EPDS ≥14 (screen-positive depression). Primary psychosocial factors were the R-AAS typology (secure, avoidant, anxious–ambivalent) and the perception of motherhood (positive, ambivalent, negative). Adjustment covariates comprised age (years), parity, pregnancy planning, and partner support; additional socio-demographic variables were explored descriptively. Bivariate analyses used χ^2^ tests (or Fisher’s exact test where appropriate), with Cramér’s V as the effect size. Multivariable analyses used binary logistic regression with non-parametric bootstrap 95% confidence intervals (B = 1000; percentile method). Penalized estimation (e.g., Firth or ridge) was considered as a robustness check in the presence of (near) complete separation; primary estimates are from the standard model. All tests were two-sided (α = 0.05). Analyses were conducted in IBM SPSS Statistics v29.

### Ethics and reporting

The study complied with the Declaration of Helsinki and national regulations on research involving human participants. Reporting follows STROBE recommendations for observational studies.

## Results

### Sample characteristics

The cohort comprised 140 third-trimester women aged 28–40 years (M = 33.56, SD = 3.19). Educational attainment was predominantly university level (73.6%), followed by upper secondary (17.9%) and doctoral (8.5%). Most participants were married (74.3%) or in a stable relationship (22.9%), and 2.8% were single mothers. Nearly half of the pregnancies were planned (48.6%), and most women perceived adequate partner support (72.9%). Perception of motherhood was positive for 20.0% of participants, ambivalent for 58.6%, and negative for 21.4%. Full sample characteristics are summarized in Table 1.

**Table 1:** Participant characteristics at third-trimester assessment

Characteristic	Category	*n*	%
Age (years)	Mean ± SD (range)	33.56 ± 3.19	28–40
Education	Upper secondary (high school)	25	17.9
	University (bachelor/master)	103	73.6
	Doctoral (PhD)	12	8.5
Marital status	Married	104	74.3
	Stable relationship (not legally married)	32	22.9
	Single mother	4	2.8
Gestational stage	Early third trimester (28–32 weeks)	86	61.4
	Late third trimester (33–39 weeks)	54	38.6
Parity	0 (primiparous)	52	37.1
	1	84	60.0
	2	4	2.9
Pregnancy planning	Planned	68	48.6
	Unplanned	72	51.4
Partner support	Present	102	72.9
	Absent	38	27.1
Perception of motherhood	Positive	28	20.0
	Ambivalent	82	58.6
	Negative	30	21.4
Attachment style (R-AAS)	Secure	26	18.6
	Avoidant	37	26.4
	Anxious–ambivalent	77	55.0

Data are *n* (%) unless otherwise stated. Age is mean ± SD (range). Early third trimester = 28–32 weeks; late third trimester = 33–39 weeks. Adult attachment categories (R-AAS) were derived from dimensional scores using the a priori decision rule described in Methods. Percentages may not sum to 100% due to rounding. Abbreviations: R-AAS, Revised Adult Attachment Scale; SD, standard deviation.

### EPDS severity and univariate associations

At the third-trimester assessment, 15.0% (21/140) screened positive for depression at EPDS ≥14. The distribution was: 0–8 = 79, 9–11 = 23, 12–13 = 17, and≥14 = 21 across categories.

**Figure 1:**
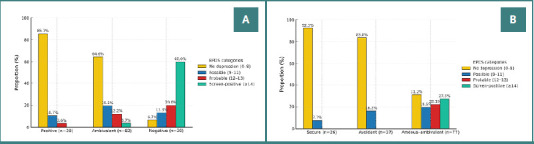
Distribution of Edinburgh Postnatal Depression Scale (EPDS) scores during the third trimester by A, perception of motherhood, and B, adult attachment style

The perception of motherhood showed a clear, graded relationship with depressive severity (χ^2^ (6) = 74.556; *P* < 0.001; Cramér’s V = 0.516). Put simply, the single perception item separated risk levels cleanly. Among women with a positive perception, almost nine in ten were in the lowest EPDS band (0–8; 85.7%, 24/28) and none reached EPDS ≥14. With an ambivalent perception, a small minority screened positive (3.7%, 3/82). In contrast, with a negative perception, about six in ten screened positive (60.0%, 18/30), and very few were symptom-minimal (0–8: 6.7%, 2/30). Panel A in Figure 1 illustrates this stepwise separation.

Attachment showed a similar pattern (χ^2^ (6) = 52.281; *P* < 0.001; Cramér’s V = 0.432). In the secure group, more than nine in ten were in the 0–8 range (92.3%, 24/26), and no cases exceeded EPDS ≥12; the avoidant group looked comparable (0–8: 83.8%, 31/37; no cases ≥12). By contrast, in the anxious–ambivalent group, over a quarter screened positive (EPDS ≥14: 27.3%, 21/77), with a corresponding shift away from the 0–8 band (31.2%, 24/77). This pattern is shown in Figure 1 (panel B). Taken together, perception (V = 0.516) separated groups even more strongly than attachment (V = 0.432), underscoring its triage value.

Exploratory crosstabs relevant to case-mix showed a modest association between parity and EPDS (χ^2^ (6) = 14.275; *P* = 0.027; Cramér’s V = 0.226) and no positive responses to EPDS item 10 at this wave. Early versus late third trimester related to attachment (χ^2^ (2) = 9.427; *P* = 0.009; V = 0.259). Pregnancy planning was associated with attachment (χ^2^ (2) = 21.258; *P* < 0.001; V = 0.390), while partner support showed a small, non-significant association with attachment (χ^2^ (2) = 4.015; *P* = 0.134; V = 0.134).

### Multivariable model (EPDS ≥14)

**Figure 2:**
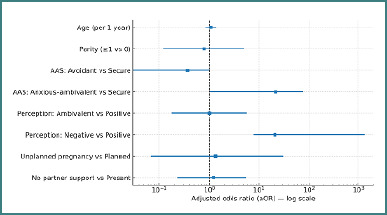
Binary logistic regression predicting screen-positive antenatal depression (EPDS ≥ 14)

In binary logistic regression (IBM SPSS, bootstrap 95% CIs) adjusted for age, parity, pregnancy planning and partner support, two factors retained independent associations with screen-positive antenatal depression: a negative perception of motherhood (adjusted odds ratio [aOR] = 21.07; 95% CI, 7.92–1317.40; reference = positive) and an anxious–ambivalent (vs secure) attachment (aOR = 21.67; 95% CI, 1.00–77.96). Other covariates were not statistically significant. Figure 2 displays the adjusted effects with non-parametric bootstrap 95% CIs (B = 1000; percentile method). Models were adjusted for age (years), parity, pregnancy planning, and partner support, reference categories in parentheses. Table 2 reports coefficients and intervals. Models adjusted for age (years), parity (≥1 vs 0), pregnancy planning (unplanned vs planned), and partner support (absent vs present). Reference categories: positive perception of motherhood; secure attachment (R-AAS); planned pregnancy; partner support present; parity = 0. Two-sided α = 0.05. Adjusted odds ratios >1 indicate higher odds relative to the stated reference.

**Table 2:** Binary logistic regression for EPDS ≥14 with bootstrap 95% confidence intervals (B = 1000; percentile method)

Variable (reference category)	aOR	95% CI
Perception: Ambivalent vs Positive	1.00	0.18–5.88
Perception: Negative vs Positive	21.07	7.92–1317.40
R-AAS: Avoidant vs Secure	0.37	0.02–1.00
R-AAS: Anxious–ambivalent vs Secure	21.67	1.00–77.96
Unplanned pregnancy vs Planned	1.33	0.07–31.19
No partner support vs Present	1.21	0.23–5.56
Parity (≥1 vs 0)	0.79	0.12–5.12
Age (per 1-year increase)	1.07	0.84–1.37

Models adjusted for age (years), parity (≥1 vs 0), pregnancy planning (unplanned vs planned), and partner support (absent vs present). Reference categories: positive perception of motherhood; secure attachment (R-AAS); planned pregnancy; partner support present; parity = 0. Two-sided α = 0.05. Adjusted odds ratios (aOR) >1 indicate higher odds relative to the stated reference. Abbreviations: EPDS, Edinburgh Postnatal Depression Scale; R-AAS, Revised Adult Attachment Scale; aOR, adjusted odds ratio; CI, confidence interval.

Clinically, the signal is concentrated in two areas—the perception item and the attachment typology—both showing large aORs > 20. The wide confidence intervals likely reflect sparse data cells and the low base rate of EPDS scores ≥14, rather than instability of the model.

### Sensitivity and robustness

Using EPDS ≥12 as the endpoint preserved directions of effect, with attenuated magnitudes and narrower intervals. An exploratory R-AAS-by-perception interaction did not indicate systematic departures from a main-effects specification, although subgroup sizes warrant caution. Penalized solutions were stable under bootstrap resampling (B = 1000); multicollinearity diagnostics were acceptable, and all resampling used a fixed random seed to support reproducibility.

## Discussion

The objective of this study was to examine whether adult attachment style and the perception of motherhood are associated with antenatal depressive severity during the third trimester.

### Main findings and interpretation

In this third-trimester cohort, depressive severity on the EPDS clustered in clear psychosocial patterns. Two signals consistently emerged as most prominent: an anxious–ambivalent attachment style and a negative perception of motherhood. Both were associated with a higher symptom burden in unadjusted comparisons and remained independently linked to screen-positive depression (EPDS ≥14) after adjusting for age, parity, pregnancy planning, and partner support. The perception item displayed a clear stepwise gradient: positive perceptions were almost exclusively associated with minimal symptoms, ambivalence indicated a modest intermediate risk, and negative perceptions accounted for most screen-positive cases. From an attachment-theory perspective, hyperactivation and heightened affective reactivity in the anxious–ambivalent pattern plausibly may increase vulnerability during the transition to motherhood, whereas secure – and, to a lesser extent, avoidant – profiles tended to co-occur with minimal symptoms [13-15].

### Relation to existing evidence and guidance

These observations sit comfortably within the broader literature. They align with meta-analytic evidence on EPDS performance [11,12] and with well-described links between social context (support networks, pregnancy intentionality) and perinatal depression [16-18]. They are also compatible with guideline frameworks (ACOG, NICE, WHO, USPSTF) that emphasize routine, repeated screening embedded in workable referral and treatment pathways [7-10]. Within that pragmatic frame, adding a brief attachment typology and a single, standardized question on the perception of motherhood appears to contribute clinically useful information for third-trimester triage, without adding substantial burden.

### Methodological considerations

We anticipated challenges typical of clinical datasets with low event rates (e.g., few EPDS ≥14 cases) and addressed them in three ways. First, we used binary logistic regression with non-parametric bootstrap 95% confidence intervals (B = 1000; percentile method) to stabilize inference under small or imbalanced cells; a fixed random seed was used to support reproducibility. Second, we considered penalized estimation (e.g., Firth or ridge) as a robustness check in the presence of (near) complete separation, while primary estimates came from the standard model. Third, we presented both distributional views (Figure 1, panels A–B) and adjusted effects (Figure 2) to aid clinical interpretation. R-AAS categories were derived from dimensional scores via transparent, median-based rules; while pragmatic, this operationalization may introduce non-differential misclassification that would tend to attenuate associations. The single-item measure for the perception of motherhood was chosen for feasibility; by design, it cannot capture nuance and warrants validation against multi-item scales. Finally, the cross-sectional design precludes temporal inference, and the urban, volunteer cohort may limit generalizability; findings should therefore be read as associations rather than causal effects.

### Clinical implications

In routine care, a brief, stepped workflow is feasible and easy to communicate to teams:

(1) Screen with EPDS.
(2) Ask one standardized question on the perception of motherhood (positive/ambivalent/negative).
(3) Classify attachment using a compact typology (secure/avoidant/anxious–ambivalent).

Used together, these three elements help flag profiles that merit a closer conversation and timely support. Clear thresholds can guide action: immediate referral to perinatal mental-health evaluation is justified for EPDS ≥14 or any endorsement of item 10; intermediate profiles – such as EPDS 9–11 accompanied by ambivalent perception or an anxious–ambivalent attachment – warrant active monitoring plus brief, evidence-based interventions (e.g., cognitive behavioral or interpersonal therapy) [10,16]. In resource-constrained services, EPDS-9 may be paired with the perception question to maintain coverage and frequency of screening. Importantly, screening initiates –not replaces – clinical assessment and feedback to women, which should be non-stigmatizing, empathic, and action-oriented.

### Strengths and limitations

Strengths include the specific focus on the third trimester; convergence across two complementary psychosocial indicators (attachment and perception of motherhood); and the use of regularized models with bootstrap inference to handle sparse cells and quantify uncertainty. Limitations include the cross-sectional design, urban sampling, the single-item perception measure, and median-based R-AAS categorization. Sparse EPDS ≥14 cells contributed to wide intervals despite penalization, reinforcing the need for replication in larger and more diverse samples.

### Future directions

Prospective work should examine the stability and incremental value of attachment and perception indicators from late pregnancy into the postpartum, including calibration and decision-analytic utility (e.g., predicted probabilities). Larger samples can test interactions (e.g., attachment × perception) and explore external validity beyond urban settings. Linking screening to service-level outcomes – acceptability, uptake, time-to-referral – would inform scale-up and help align triage thresholds with real-world capacity.

## Conclusion

In late pregnancy, anxious–ambivalent attachment and a negative perception of motherhood were independently associated with screen-positive depressive symptoms (EPDS ≥ 14), whereas socio-demographic factors were not. Combining a brief attachment assessment and a single perception-of-motherhood item with EPDS may improve psychosocial risk identification in antenatal care. These low-burden, non-pharmacological screening tools can support early conversation and referral within obstetric–rehabilitation settings.

## Data Availability

De-identified data underlying this study are available from the corresponding author upon reasonable request. Data are not publicly available due to participant privacy and ethics restrictions. This manuscript reports analyses from the third-trimester wave (Stage 3) of a longitudinal cohort. A separate manuscript reports findings from a Stage 4 subsample involving oxytocin quantification; objectives, endpoints, and statistical models are distinct, and there is no text reuse between manuscripts.

## Personal thanks

We thank the participants for their time and commitment.
